# Changing Practice Patterns and Improving Survival for Patients with Pancreatic Ductal Adenocarcinoma

**DOI:** 10.3390/cancers15184464

**Published:** 2023-09-07

**Authors:** Patrick W. Underwood, Kelly M. Herremans, Dan Neal, Andrea N. Riner, Ibrahim Nassour, Steven J. Hughes, Jose G. Trevino

**Affiliations:** 1 Department of Surgery, College of Medicine, University of Florida, Gainesville, FL 32610, USA; patrick.underwood@surgery.ufl.edu (P.W.U.); kelly.herremans@surgery.ufl.edu (K.M.H.); dneal@ufl.edu (D.N.); andrea.riner@surgery.ufl.edu (A.N.R.); ibrahim.nassour@surgery.ufl.edu (I.N.); steven.hughes@surgery.ufl.edu (S.J.H.); 2 Department of Surgery, School of Medicine, Virginia Commonwealth University, Richmond, VA 23298, USA

**Keywords:** pancreatic ductal adenocarcinoma, National Cancer Database, survival patterns, treatment

## Abstract

**Simple Summary:**

There have been a number of advances in the treatment paradigm of pancreatic ductal adenocarcinoma (PDAC) over the past two decades. Medical and surgical approaches to PDAC have continued to evolve. The individual effects of these changes have been investigated. Understanding the implementation of these changes over time and its effect on patient survival is important. While patient survival from PDAC has been reported to have modest improvements over the decades, the National Cancer Database offers the ability to study survival trends. We aim to evaluate survival trends in patients diagnosed with PDAC in the United States.

**Abstract:**

Over the last two decades, there have been many reported advances in the clinical management of pancreatic ductal adenocarcinoma (PDAC). We sought to evaluate changes in survival for patients diagnosed with PDAC between 2004 and 2017. The National Cancer Database was queried for patients diagnosed with PDAC between 2004 and 2017. There were 55,401 patients who underwent surgery and 109,477 patients who underwent non-surgical treatment for PDAC between 2004 and 2017. Patients were categorized into four groups by year of diagnosis. Median survival improved from 15.5 months to 25.3 months for patients treated with surgery between the years 2016 and 2017 compared with between 2004 and 2007 (*p* < 0.001). Median survival improved from 7.2 months to 10.1 months for patients treated without surgery during the same years (*p* < 0.001). On multivariable analysis, the hazard ratio for death was estimated to multiply by 0.975 per year for patients treated with surgery and 0.959 per year for patients treated without surgery (*p* < 0.001). This increase in survival in the setting of evolving care validates continued efforts aimed at improving survival for patients with this devastating disease.

## 1. Introduction

Pancreatic ductal adenocarcinoma (PDAC) remains a deadly malignancy with an 11% five-year survival [1]. Only 15–20% of patients undergo potentially curative surgical resection [2]. Even after resection, five-year survival remains a dismal 24% [3]. For those unable to undergo surgical resection, survival is a mere 3–14 months [1]. In order to improve survival for these patients, PDAC remains the subject of intense scientific investigation. Unfortunately, despite these efforts, there has been relatively little increase in overall survival [1,4].

In the last two decades, there have been significant changes in the treatment paradigm of PDAC. Neoadjuvant chemotherapy has become more commonplace for even resectable PDAC [5]. The chemotherapy regimens used, whether neoadjuvant, adjuvant, or for unresectable disease, have changed [6,7]. Meanwhile, surgical treatment for PDAC has evolved as mounting evidence has shown that pancreatic resections should be performed at high volume centers [8,9], minimally invasive pancreatectomies have been shown to have similar outcomes to open procedures [10], and more aggressive vascular resections have increased in frequency [11]. Enhanced recovery after surgery (ERAS) protocols have improved outcomes with the goal of safely and efficiently recovering patients after surgery [12].

The effect of many of these changes on survival for patients with PDAC has been individually evaluated and, while there are likely numerous factors not listed here that have further contributed to patient outcomes, the overall trends in survival for patients treated for PDAC have not been well described. We aimed to use the National Cancer Database (NCDB) to evaluate changes in survival for patients diagnosed with pancreatic ductal adenocarcinoma between 2004 and 2017.

## 2. Materials and Methods

### 2.1. Data and Study Population

The NCDB participant user file for patients with pancreatic malignancies diagnosed between 2004 and 2017 was queried for patients with pancreatic ductal adenocarcinoma (PDAC). The NCDB is a joint project of the Commission on Cancer of the American College of Surgeons and the American Cancer Society. It includes data from more than 1500 commission-accredited cancer programs in the United States. The data used in the study are derived from a deidentified NCDB file. The American College of Surgeons and the Commission on Cancer have not verified, and are not responsible for, the analytic or statistical methodology employed or the conclusions drawn from these data by the investigator. The database includes about 70% of all newly diagnosed cancers in the United States [13]. Because the data are deidentified, they are IRB exempt. There were 281,900 cases. Additional exclusion criteria included no treatment (medical or surgical), missing survival data, or missing surgery data. The final study population for analysis included 164,878 patients. 

### 2.2. Variables and Outcomes

Patient characteristics and socioeconomic variables included facility type and location, age, sex, race, ethnicity, insurer, median income and percentage of population without a high school degree for the ZIP code in which the patient resides, distance from hospital, rural/urban residence, and Charlson–Deyo comorbidity index. Oncologic factors included tumor location, tumor size, stage, days from diagnosis to treatment, lymph nodes, margin status, neoadjuvant therapy, receipt of chemotherapy, readmission, and mortality. Survival data were available for patients between 2004 and 2017. Patients were grouped into four groups by year of diagnosis: 2004–2007, 2008–2011, 2012–2015, 2016–2017. The primary outcome of interest was survival. In patients undergoing surgery, survival was defined as time from surgery to death or most recent follow-up. In patients who did not undergo surgery, survival was defined as time from diagnosis until death of most recent follow-up.

### 2.3. Statistical Analysis

Statistical analysis was conducted using the R statistical software package (V.3.6.3, The R Foundation for Statistical Computing, Vienna, Austria). Descriptive statistics by time period were created for the variables of interest. Since interest was in assessing trends over time, the time periods were treated as ordered categories and associations with time were assessed using Spearman correlation tests (continuous variables) or logistic regression models (categorical variables), with the factor as outcome and the time period as predictor. Kaplan–Meier methods were used for estimating survival by year of diagnosis.

Mixed-effects Cox regression was used to estimate the effect of year of diagnosis (treated as a continuous variable) on survival when controlling for patient and hospital factors. Surgical and non-surgical patients were analyzed in separate models. A random effect for hospital was included in both models to adjust for the clustering of observations on center. Variables missing ≤5% of cases were imputed with mean, median, or mode. Variables missing in >5% but <10% of cases were not imputed; instead, a “missing” category was added to these variables. Tumor size and postoperative length of stay were excluded due to >10% missingness. For surgical patients, fixed factors were year of diagnosis, facility type, facility location, age, sex, race, ethnicity, insurer, median income and percentage of population without a high school degree for the zip code in which the patient resides, distance from hospital, rural/urban residence, Charlson–Deyo comorbidity index, primary site, histology, stage, number of lymph nodes examined, number of nodes positive, receipt of chemotherapy, T stage, N stage, and days from diagnosis to first treatment. For non-surgical patients, fixed factors were year, facility type, facility location, age, sex, race, ethnicity, insurer, median income and percentage of population without a high school degree for the zip code in which the patient resides, distance from hospital, urban/rural residence, Charlson–Deyo comorbidity index, primary site, histology, stage, number of nodes examined, number of nodes positive, receipt of chemotherapy, and days from diagnosis to first treatment. For both the surgical patient and non-surgical patient modeling, the natural log of distance, nodes examined, and nodes positive were used in the model because these variables were highly skewed. In order to generate survival curves using the Cox regression model, we selected a patient with the average characteristics of the study population. The average characteristics were represented by either the mean or mode, depending on whether the variable was categorical or continuous. 

## 3. Results

### 3.1. Surgical Patient Characteristics

There were 55,401 patients who underwent surgical resection for PDAC between 2004 and 2017 (Table 1). Average age at diagnosis increased minimally from 2004 to 2007 to 2016 to 2017 (65.0 ± 10.9 years to 65.8 ± 10.0 years, *p* < 0.001). The sex of patients did not significantly change (*p* = 0.072). As the percentage of patients being treated at an academic center decreased from 52.4 to 49.3% (*p* < 0.001), the rate of patients treated at a comprehensive community cancer center increased from 25.9 to 27.4% (*p* < 0.001). The rate of patients insured by Medicare increased from 49.7 to 53.3% (*p* < 0.001) and the percentage of privately insured patients decreased from 42.7 to 37.8% (*p* < 0.001). The percentage of patients living in zip codes with median income <USD 38,000 increased from 12.8 to 16.2% (*p* < 0.001) and patients living in zip codes with median income >USD 63,000 decreased from 41.7 to 38.7% (*p* < 0.001). Patients living in zip codes where >21% of the population had not achieved high school education increased from 15.1 to 18.5% (*p* < 0.001). Patients living in zip codes where < 7% of the population had not achieved high school education decreased from 37.5 to 27.0% (*p* < 0.001).

Patients with a Charlson–Deyo comorbidity index score of 0 decreased from 68.1 to 64.6% (*p* < 0.001), while Charlson–Deyo comorbidity index scores >3 rose from 1.8 to 4.8% (*p* < 0.001). The vast majority of patients had stage 1 or stage 2 disease across all years. The number of examined lymph nodes increased from 11.7 ± 8.5 to 18.9 ± 10.4 (*p* < 0.001) The percentage of patients receiving chemotherapy increased from 63.2 to 81.7% (*p* < 0.001). Length of stay decreased from a median of 9 days to a median of 7 days. Mortality at 30 days, 90 days, 1 year, and 2 years all decreased significantly (*p* < 0.001).

### 3.2. Survival after Surgery

**Figure 1:**
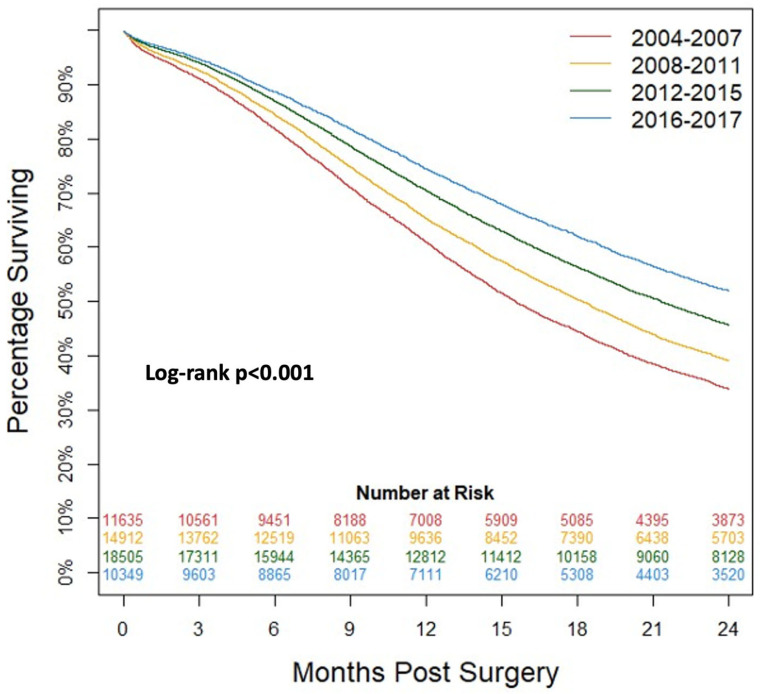
Kaplan–Meier analysis of patients undergoing surgery for pancreatic ductal adenocarcinoma grouped by year of diagnosis.

Kaplan–Meier analysis of survival in patients undergoing surgery for PDAC was performed (Figure 1). Patients were again grouped into four groups by year of diagnosis (2004–2007, 2008–2011, 2012–2015, 2016–2017). There was a significant increase in patient survival across years (*p* < 0.001). Table 2 shows that the percentage of patients alive at all time intervals through 24 months increased significantly (*p* < 0.001). Median survival increased from 15.5 (95% confidence interval (CI): 15.2–15.9) months to 25.3 (95%CI: 24.7–26.1) months from the years 2004 to 2007 to 2016 to 2017.

**Figure 2:**
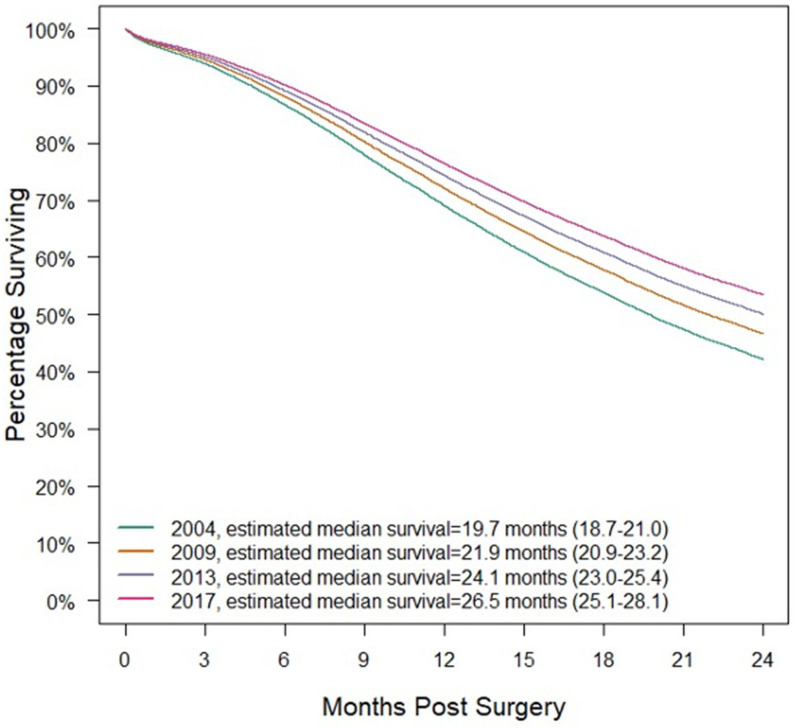
Risk-adjusted survival by year for the average surgical patient with median survival and 95%CIs. The average surgical patient is defined in the results section.

Controlling for other variables as outlined in the methods, the hazard ratio for death is estimated to multiply by 0.975 (95%CI: 0.971–0.981, *p* < 0.001) per year. Hazard ratios for death by stage can be found in Supplementary Materials Table S1. The hazard ratios for other variables in the Cox regression can be found in Supplementary Materials Table S2. Figure 2 demonstrates risk-adjusted survival by year for the average surgical patient. The average surgical patient was a hypothetical patient with the following characteristics: a 65-year-old non-Hispanic White male with a Charlson–Deyo Score of 0, insured by Medicare, and lived in a metro area 17 miles from the hospital in the Mid-Atlantic region, with a zip code with the highest income quartile and 6.3–10.8% of residents without a high school education. This hypothetical patient had a stage II pancreatic head malignancy who began treatment 16 days after diagnosis at an academic center and was found to have 3/16 lymph nodes positive, with final pathologic stage pT3N1, for which chemotherapy was also administered. For an average patient diagnosed with PDAC in 2004 and undergoing surgery, median survival is estimated at 19.7 (95%CI: 18.7–21.0) months. For an average patient diagnosed with PDAC in 2017 and undergoing surgery, median survival is estimated at 26.5 (95%CI: 25.1–28.1) months.

### 3.3. Non-Surgical Patient Characteristics

There were 109,477 patients who received non-operative treatment for pancreatic ductal adenocarcinoma between 2004 and 2017 (Table 3). Age at diagnosis again increased minimally from 2004 to 2007 (65.2 ± 11.2 years to 65.9 ± 10.4 years) and the sex of patients did not significantly change (*p* = 0.095). In contrast to surgical patients, the rate of patients receiving treatment at an academic center increased from 39.2 to 42% (*p* < 0.001) and the rate of patients being treated at a comprehensive community cancer center decreased from 35.4 to 33.5% (*p* < 0.001). Patients insured by Medicare increased from 48.1 to 53.4% (*p* < 0.001), whereas privately insured patients decreased from 43.5 to 35.6% (*p* < 0.001). The percentage of patients living in zip codes with median income <USD 38,000 increased from 12.8 to 17.6% (*p* < 0.001). The percentage of patients living in zip codes with median income >USD 63,000 decreased from 42.4 to 37.8% (*p* < 0.001). Patients living in zip codes with a percentage of the population with less than high school education of >21% increased from 15.4 to 19.7% (*p* < 0.001). Patients living in zip codes with a percentage of the population with less than high school education of <7% decreased from 37.7 to 26.0% (*p* < 0.001).

### 3.4. Survival after Non-Surgical Treatment

**Figure 3:**
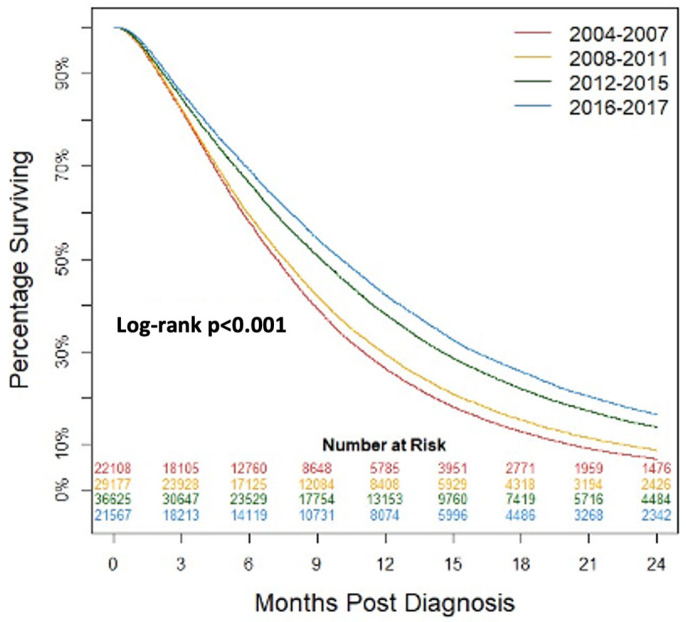
Kaplan–Meier analysis of patients undergoing non-surgical treatment for pancreatic ductal adenocarcinoma grouped by year of diagnosis.

Kaplan–Meier analysis of survival in patients treated medically for PDAC was performed (Figure 3). There was a significant increase in patient survival across years (*p* < 0.001). Table 4 shows the percentage of patients alive at all time intervals through 24 months increased significantly (*p* < 0.001). Median survival increased from 7.2 (95%CI: 7.1–7.3) months to 10.1 (95%CI: 9.9–10.2) months from the years 2004 to 2007 to 2016 to 2017.

**Figure 4:**
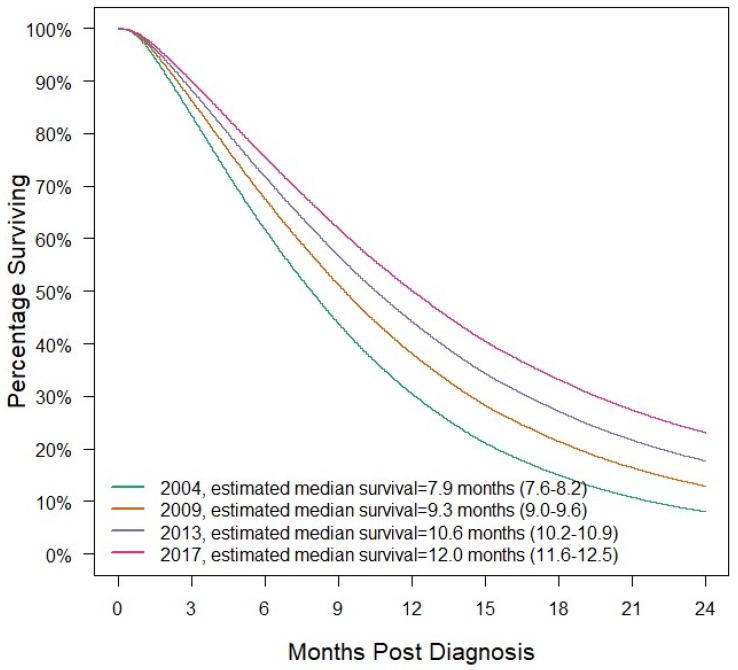
Risk-adjusted survival by year for the average non-surgical patient with median survival and 95%CIs. The average non-surgical patient is defined in the results section.

After adjusting for covariates, the hazard ratio for death is estimated to multiply by 0.959 (95%CI: 0.957–0.962, *p* < 0.001) per year. Hazard ratios for death by stage can be found in Supplementary Materials Table S3. The hazard ratios for other variables in the Cox regression can be found in Supplementary Materials Table S4. Figure 4 demonstrates risk-adjusted survival by year for the average patient treated medically. The average patient treated non-operatively was a hypothetical patient with the following characteristics: a 65-year-old non-Hispanic White male with Charlson–Deyo Score of 0, insured by Medicare, and lived in a metro area 17 miles from the hospital in the Mid-Atlantic region, with a zip code with the highest income quartile and 6.3–10.8% of residents without a high school education. This hypothetical patient had a pancreatic head malignancy with stage 4 disease and began treatment 27 days after diagnosis at an academic center. For an average patient diagnosed with PDAC in 2004 and treated medically, median survival is estimated at 7.9 (95%CI: 7.6–8.2) months. For an average patient diagnosed with PDAC in 2017 and treated medically, median survival is estimated at 12 (95%CI: 11.6–12.5) months.

## 4. Discussion

There is a lack of recent data on a national level evaluating changes in survival for patients undergoing surgical and non-surgical management of PDAC. This study is an in-depth analysis of survival in patients from 2004 to 2017. The results of this study indicate that survival for patients with PDAC has improved significantly in recent years, most impressively for patients who underwent surgical resection. This patient population experienced a 10-month increase in median survival over the study time interval. This is in contrast to an improvement in survival of only about 3 months for patients treated without surgery.

Several studies have noted increased incidence and mortality of pancreatic cancer over time. A study of the Surveillance, Epidemiology, and End Results (SEER) database found pancreatic cancer incidence increased 1.03% per year between 1974 and 2014. In the same study, mortality increased 2.22% per year [14]. Another study of the SEER database found that PDAC incidence and mortality are likely to continue to increase and become the second leading cause of cancer-related death [15]. A similar study using the SEER database between 1981 and 2010 found an increase in the 24-month survival rate from 6.7 to 14% when comparing patients diagnosed between 1981 and 1990 compared with between 2001 and 2010 [16]. This is similar to the 24-month survival rates found in our study. An analysis of multiple national cancer registries compared survival trends over time. The SEER database was used as the United States of America cancer registry. In stage I-II patients, it found an increase in 36-month survival from 13 to 17% when comparing 2003 to 2005 to 2009 to 2011. In patients with stage III–IV disease, the survival increase was <1% [17]. Our study is the first recent analysis of survival trends over timing using the NCDB which captures significantly more cases compared with the SEER registry (70% vs. 28%) [13]. The findings presented here demonstrate that survival has continued to increase since these studies.

Surgical outcomes improved in our study era, including a 2.3-day reduction in hospital length of stay. This finding may be attributable to ERAS protocols and minimally invasive pancreatic resections, which have become more common. Multiple studies have shown that ERAS protocols can lead to decreased length of stay, with similar readmission rates for patients undergoing pancreas surgery [18]. Any effect these had on length of stay in our study would be speculative. Chemotherapy has also evolved for the surgical patient. FOLFIRINOX regimens have come into favor over gemcitabine for patients with metastatic disease and in the adjuvant setting due to a significant survival benefit [6,7]. During the study timeframe, we demonstrated a significant increase in neoadjuvant therapy usage from 7.1 to 32.4% for patients treated with surgery. This is not surprising, as many centers now favor neoadjuvant therapy for nearly all patients, including resectable PDAC [19,20]. The number of patients treated with neoadjuvant therapy will likely continue to grow. The rate of R0 resection (microscopically margin negative) also increased during this timeframe. Previous studies investigating neoadjuvant chemotherapy have shown an increase in R0 resection for patients with resectable PDAC who are treated with neoadjuvant chemotherapy but with no clear survival benefit [21]. The survival benefit in surgical patients in this study likely reflects improvements in not only surgical care of patients but also multidisciplinary advances in the management of PDAC and perioperative care.

For patients treated without surgery, there were significant increases in survival in both Kaplan–Meier analysis and multivariable analysis. The majority of these patients had locally advanced or metastatic disease at diagnosis. This diagnosis has poor prognosis and the results of our study suggest there is much work to be conducted for improving survival for these patients. Results of trials using FOLFIRINOX regimens were published during our study period and have demonstrated improvement in survival for patients with advanced disease [7,22]. It is possible that this is contributing to the increased survival seen in our study population. Aside from this change, there have been no paradigm shifting treatments for unresectable or metastatic PDAC. Effective immunotherapies have not been discovered, although this remains an area of ongoing investigation [23,24]. Given that the majority of patients with PDAC present with unresectable or metastatic disease [25], new therapeutic regimens represent the greatest opportunity to move the needle in overall survival for patients with PDAC.

This retrospective review of a national dataset carries a number of limitations. First, the NCDB captures 70% of newly diagnosed cancers [13]; this leaves about 30% of patients who are not captured in this database. The treatment and survival of these patients is unknown. Although the sample size is large, there is limited granular data to assess the factors contributing to the observed survival differences. About 5% of patients who underwent “surgery of the primary site” were classified as having stage IV disease. If properly classified according to the NCDB data dictionary, this category should not include patients who underwent diagnostic laparoscopy, palliative surgery, or other surgery that did not include removal of the tumor. The reason these patients would have undergone resection is unclear but may represent miscoding. The degree of miscoding for other variables is unknown. Exact treatment, such as chemotherapy regimens, are unable to be determined. Despite these limitations, the large sample size and overall results of the study carry importance.

## 5. Conclusions

The results of our study suggest improving survival for patients with PDAC whether treated with surgery or medical therapy. Future studies to understand which factors are responsible for improved survival are warranted. While these data validate the efforts of physicians and scientist, they also demonstrate a significant need for continued work to improve outcomes for patients. Nevertheless, this overall improvement in survival is a significant achievement towards better helping patients with this deadly disease.

## Figures and Tables

**Table 1:** Surgical patient characteristics.

	Overall (N = 55,401)	2004–2007 (N = 11,635, 21.0%)	2008–2011 (N = 14,912, 26.9%)	2012–2015 (N = 18,505, 33.4%)	2016–2017 (N = 10,349, 18.7%)	*p* (Trend)	% Miss.
Age	65.4 ± 10.4	65.0 ± 10.9	65.4 ± 10.6	65.5 ± 10.3	65.8 ± 10.0	<0.0001	0
Female	27,060 (48.8)	5740 (49.3)	7336 (49.2)	8979 (48.5)	5005 (48.4)	0.072	0
Race							
White	47,290 (86.2)	10,052 (87.5)	12,810 (86.7)	15,716 (85.6)	8712 (84.9)	<0.0001	0.9
Black	5498 (10.0)	1101 (9.6)	1462 (9.9)	1909 (10.4)	1026 (10.0)	0.108	
Other	2092 (3.8)	330 (2.9)	505 (3.4)	737 (4.0)	520 (5.1)	<0.0001	
Hispanic origin	2824 (5.3)	507 (4.8)	683 (4.8)	969 (5.3)	665 (6.5)	<0.0001	4.3
Distance from hospital ^2^	17 [6, 46]	16 [6, 45]	16 [6, 45]	17 [7, 47]	17 [7, 45]	<0.0001	6.7
Facility type							
Community	1654 (3.0)	418 (3.6)	401 (2.7)	462 (2.5)	373 (3.6)	0.412	0.9
Comp. comm.	14,283 (26.0)	2978 (25.9)	3675 (24.9)	4823 (26.3)	2807 (27.4)	0.0009	
Academic	28,591 (52.1)	6038 (52.4)	7847 (53.1)	9650 (52.6)	5056 (49.3)	<0.0001	
Int. network	10,349 (18.9)	2082 (18.1)	2846 (19.3)	3408 (18.6)	2013 (19.6)	0.034	
Facility Location							
New England	2496 (4.5)	533 (4.6)	632 (4.3)	821 (4.5)	510 (5.0)	0.191	0.9
Middle Atlantic	9194 (16.8)	1921 (16.7)	2559 (17.3)	3081 (16.8)	1633 (15.9)	0.089	
South Atlantic	12,110 (22.1)	2536 (22.0)	3267 (22.1)	4016 (21.9)	2291 (22.4)	0.747	
East N. Central	9720 (17.7)	2079 (18.1)	2654 (18.0)	3261 (17.8)	1726 (16.8)	0.024	
East S. Central	3824 (7.0)	811 (7.0)	1097 (7.4)	1325 (7.2)	591 (5.8)	0.0006	
West N. Central	4356 (7.9)	983 (8.5)	1241 (8.4)	1382 (7.5)	750 (7.3)	<0.0001	
West S. Central	4998 (9.1)	1009 (8.8)	1261 (8.5)	1744 (9.5)	984 (9.6)	0.002	
Mountain	2142 (3.9)	427 (3.7)	526 (3.6)	708 (3.9)	481 (4.7)	0.0001	
Pacific	6037 (11.0)	1217 (10.6)	1532 (10.4)	2005 (10.9)	1283 (12.5)	<0.0001	
Insurer							
Medicare	27,876 (51.2)	5611 (49.7)	7393 (50.3)	9421 (51.7)	5451 (53.3)	<0.0001	1.8
Private	21,653 (39.8)	4827 (42.7)	5986 (40.7)	6977 (38.3)	3863 (37.8)	<0.0001	
Medicaid	2790 (5.1)	433 (3.8)	720 (4.9)	1074 (5.9)	563 (5.5)	<0.0001	
Other Government	740 (1.4)	109 (1.0)	179 (1.2)	269 (1.5)	183 (1.8)	<0.0001	
None	1366 (2.5)	315 (2.8)	413 (2.8)	473 (2.6)	165 (1.6)	<0.0001	
Income quartile							
<USD 40,227	7958 (15.4)	1457 (12.8)	2236 (15.6)	2842 (16.6)	1423 (16.2)	<0.0001	6.8
USD 40,277–USD 50,353	10,965 (21.2)	2115 (18.6)	3062 (21.3)	3899 (22.8)	1889 (21.5)	<0.0001	
USD 50,354–USD 63,332	13,343 (25.8)	3057 (26.9)	3828 (26.7)	4388 (25.6)	2070 (23.6)	<0.0001	
>USD 63,333	19,374 (37.5)	4750 (41.7)	5220 (36.4)	6008 (35.1)	3396 (38.7)	<0.0001	
Education quartile							
>17.6%	8480 (16.4)	1713 (15.1)	2256 (15.7)	2887 (16.8)	1624 (18.5)	<0.0001	6.8
10.9–17.5%	12,879 (24.9)	2672 (23.5)	3577 (24.9)	4424 (25.8)	2206 (25.1)	0.0005	
6.3–10.8%	15,200 (29.4)	2734 (24.0)	4413 (30.8)	5468 (31.9)	2585 (29.4)	<0.0001	
<6.3%	15,100 (29.2)	4263 (37.5)	4104 (28.6)	4365 (25.5)	2368 (27.0)	<0.0001	
Metro area							
Metro	44,279 (83.1)	9104 (81.2)	11,939 (82.9)	14,837 (83.5)	8399 (84.6)	<0.0001	3.8
Metro adjacent	5743 (10.8)	1279 (11.4)	1606 (11.2)	1862 (10.5)	996 (10.0)	0.0002	
Non-metro adj.	2259 (4.2)	565 (5.0)	586 (4.1)	736 (4.1)	372 (3.7)	<0.0001	
Rural	1014 (1.9)	260 (2.3)	268 (1.9)	324 (1.8)	162 (1.6)	0.0004	
Charlson–Deyo Score							
0	36,612 (66.1)	7918 (68.1)	9923 (66.5)	12,083 (65.3)	6688 (64.6)	<0.0001	0
1	14,017 (25.3)	2877 (24.7)	3807 (25.5)	4884 (26.4)	2449 (23.7)	0.503	
2	3333 (6.0)	634 (5.4)	880 (5.9)	1104 (6.0)	715 (6.9)	<0.0001	
3+	1439 (2.6)	206 (1.8)	302 (2.0)	434 (2.3)	497 (4.8)	<0.0001	
Primary site							
Head of pancreas	40,280 (72.7)	8522 (73.2)	10,856 (72.8)	13,432 (72.6)	7470 (72.2)	0.071	0
Body of pancreas	3772 (6.8)	656 (5.6)	910 (6.1)	1373 (7.4)	833 (8.0)	<0.0001	
Tail of pancreas	5216 (9.4)	964 (8.3)	1353 (9.1)	1804 (9.7)	1095 (10.6)	<0.0001	
Pancreatic duct	412 (0.7)	141 (1.2)	117 (0.8)	99 (0.5)	55 (0.5)	<0.0001	
Islets of Langerhans	5 (0)	2 (0)	2 (0)	0 (0)	1 (0)	NA	
Other	548 (1.0)	74 (0.6)	146 (1.0)	196 (1.1)	132 (1.3)	<0.0001	
Overlapping lesion of pancreas	2223 (4.0)	428 (3.7)	623 (4.2)	775 (4.2)	397 (3.8)	0.461	
Pancreas, NOS	2945 (5.3)	848 (7.3)	905 (6.1)	826 (4.5)	366 (3.5)	<0.0001	
Neoadjuvant therapy	9633 (19.5)	435 (7.1)	1856 (12.6)	4011 (21.8)	3331 (32.4)	<0.0001	10.7
Tumor size ^1^	31 [25, 40]	31 [25, 41]	31 [25, 40]	32 [25, 40]	32 [25, 40]	0.777	20.2
R1 Resection	12,512 (23.6)	2975 (27.1)	3513 (24.7)	4023 (22.6)	2001 (20.0)	<0.0001	4.2
Stage							
1	6553 (12.2)	1492 (13.8)	1765 (12.2)	1936 (10.6)	1360 (13.2)	0.0008	2.9
2	42,233 (78.5)	7906 (73.4)	11,285 (78.2)	14,916 (81.4)	8126 (79.1)	<0.0001	
3	2295 (4.3)	637 (5.9)	606 (4.2)	704 (3.8)	348 (3.4)	<0.0001	
4	2722 (5.1)	739 (6.9)	781 (5.4)	758 (4.1)	444 (4.3)	<0.0001	
Treatment started, days from diagnosis	21.1 ± 27.1	17.1 ± 23.7	19.9 ± 33.1	22.4 ± 24.7	24.9 ± 24.4	<0.0001	1.9
Nodes examined	15.8 ± 10.2	11.7 ± 8.5	15.2 ± 9.9	17.2 ± 10.4	18.9 ± 10.4	<0.0001	1.7
Nodes positive	2.4 ± 3.2	2.0 ± 2.9	2.4 ± 3.2	2.5 ± 3.3	2.5 ± 3.4	<0.0001	1.2
Received chemotherapy	39,075 (73.8)	6947 (63.2)	10,085 (71.4)	13,864 (77.8)	8179 (81.7)	<0.0001	4.4
Postop LOS ^1^	8 [6, 12]	9 [7, 14]	9 [6, 13]	8 [6, 12]	7 [5, 10]	<0.0001	12.6
30-day unplanned readmission	3843 (7.2)	806 (7.3)	1106 (7.7)	1262 (7.0)	669 (6.6)	0.005	3.1
30-day mortality	1783 (3.2)	497 (4.3)	520 (3.5)	517 (2.8)	249 (2.4)	<0.0001	0.3
90-day mortality	3769 (6.9)	1033 (8.9)	1096 (7.4)	1099 (6.0)	541 (5.3)	<0.0001	0.7
1-year mortality	17,587 (32.5)	4502 (39.1)	5132 (34.8)	5419 (29.7)	2534 (26.3)	<0.0001	2.3
2-year mortality	30,913 (59.3)	7598 (66.2)	8983 (61.2)	9871 (54.8)	4461 (55.9)	<0.0001	5.9

^1^ For continuous variables, the “±” represents standard deviation. ^2^ median (IQR), Spearman correlation. Comp. comm.—comprehensive community cancer center, Int. network—integrated network cancer program, N.—North, S.—South, NA—Not applicable.

**Table 2:** Percent survival for surgical patients at different time intervals.

	2004–2007 (N = 11,635, 21.0%)	2008–2011 (N = 14,912, 26.9%)	2012–2015 (N = 18,505, 33.4%)	2016–2017 (N = 10,349, 18.7%)
3 months	91.1% (90.6–91.6%)	92.6% (92.2–94.1%)	94.0% (93.7–94.4%)	94.7% (94.3–95.2%)
6 months	81.8% (81.1–82.5%)	84.5% (83.9–85.1%)	87.0% (86.5–87.5%)	88.7% (88.1–89.3%)
9 months	71.0% (70.2–71.8%)	74.8% (74.1–75.5%)	78.7% (78.1–79.2%)	81.8% (81.1–82.6%)
12 months	61.0% (60.1–61.9%)	65.4% (64.6–66.1%)	70.4% (69.8–71.1%)	74.5% (73.7–75.4%)
18 months	44.5% (43.6–45.4%)	50.4% (49.6–51.2%)	56.4% (55.7–57.1%)	62.1% (61.1–63.1%)
24 months	34.0% (33.1–34.8%)	39.1% (38.3–39.9%)	45.7% (45.0–46.5%)	52.0% (50.9–53.0%)
Median survival (months)	15.5 (15.2–15.9)	18.2 (17.8–18.6)	21.3 (21.0–21.7)	25.3 (24.7–26.1)

**Table 3:** Non-surgical patient characteristics.

	Overall (N = 109,477)	2004–2007 (N = 22,108, 20.2%)	2008–2011 (N = 29,177, 26.7%)	2012–2015 (N = 36,625, 33.4%)	2016–2017 (N = 21,567, 19.7%)	*p* (Trend)	% Miss.
Age	65.2 ± 10.8	64.6 ± 11.2	65.0 ± 11.0	65.4 ± 10.6	65.9 ± 10.4	<0.0001	0
Female	51,679 (47.2)	10,507 (47.5)	13,857 (47.5)	17,191 (46.9)	10,124 (46.9)	0.095	0
Race							
White	90,257 (83.2)	18,558 (85.0)	24,241 (83.9)	29,958 (82.5)	17,500 (81.8)	<0.0001	1.0
Black	14,070 (13.0)	2619 (12.0)	3683 (12.8)	4883 (13.4)	2885 (13.5)	<0.0001	
Other	4108 (3.8)	665 (3.0)	955 (3.3)	1473 (4.1)	1015 (4.7)	<0.0001	
Hispanic origin	5706 (5.5)	922 (4.6)	1467 (5.4)	1974 (5.5)	1343 (6.3)	<0.0001	4.9
Distance from hospital ^1^	11 [5, 28]	10 [4, 26]	11 [5, 28]	12 [5, 28]	12 [5, 28]	<0.0001	4.8
Facility type							
Community	6008 (5.5)	1211 (5.5)	1569 (5.4)	1999 (5.5)	1229 (5.8)	0.308	1.0
Comp. comm.	37,122 (34.3)	7736 (35.4)	9991 (34.6)	12,243 (33.8)	7152 (33.5)	<0.0001	
Academic	44,752 (41.3)	8574 (39.2)	11,763 (40.8)	15,437 (42.6)	8978 (42.0)	<0.0001	
Int. network	20,449 (18.9)	4332 (19.8)	5543 (19.2)	6579 (18.1)	3995 (18.7)	<0.0001	
Facility Location							
New England	6545 (6.0)	1416 (6.5)	1652 (5.7)	2194 (6.1)	1283 (6.0)	0.184	1.0
Middle Atlantic	18,240 (16.8)	3686 (16.9)	4930 (17.1)	6052 (16.7)	3572 (16.7)	0.399	
South Atlantic	22,915 (21.2)	4478 (20.5)	6079 (21.1)	7741 (21.3)	4617 (21.6)	0.002	
East N. Central	19,738 (18.2)	4170 (19.1)	5260 (18.2)	6595 (18.2)	3713 (17.4)	<0.0001	
East S. Central	6825 (6.3)	1338 (6.1)	1794 (6.2)	2312 (6.4)	1381 (6.5)	0.092	
West N. Central	9308 (8.6)	2015 (9.2)	2651 (9.2)	3002 (8.3)	1640 (7.7)	<0.0001	
West S. Central	8334 (7.7)	1605 (7.3)	2267 (7.9)	2760 (7.6)	1702 (8.0)	0.066	
Mountain	4666 (4.3)	825 (3.8)	1242 (4.3)	1613 (4.4)	986 (4.6)	<0.0001	
Pacific	11,760 (10.9)	2320 (10.6)	2991 (10.4)	3989 (11.0)	2460 (11.5)	0.0002	
Insurer							
Medicare	53,528 (50.1)	10,337 (48.1)	13,731 (48.4)	18,112 (50.5)	11,348 (53.4)	<0.0001	2.3
Private	42,068 (39.3)	9339 (43.5)	11,518 (40.6)	13,647 (38.1)	7564 (35.6)	<0.0001	
Medicaid	6902 (6.5)	973 (4.5)	1825 (6.4)	2481 (6.9)	1623 (7.6)	<0.0001	
Other Government	1228 (1.1)	172 (0.8)	316 (1.1)	477 (1.3)	263 (1.2)	<0.0001	
None	3211 (3.0)	648 (3.0)	988 (3.5)	1114 (3.1)	461 (2.2)	<0.0001	
Income quartile							
<USD 40,227	16,588 (15.9)	2789 (12.8)	4607 (16.1)	5917 (16.8)	3275 (17.6)	<0.0001	4.9
USD 40,277-USD 50,353	21,736 (20.9)	3755 (17.3)	6251 (21.9)	7832 (22.3)	3898 (20.9)	<0.0001	
USD 50,354-USD 63,332	27,388 (26.3)	5981 (27.5)	7735 (27.1)	9252 (26.3)	4420 (23.7)	<0.0001	
>USD 63,333	38,420 (36.9)	9226 (42.4)	9999 (35.0)	12,145 (34.6)	7050 (37.8)	<0.0001	
Education quartile							
>17.6%	17,772 (17.1)	3347 (15.4)	4696 (16.4)	6055 (17.2)	3674 (19.7)	<0.0001	4.8
10.9–17.5%	25,376 (24.4)	4874 (22.4)	7057 (24.7)	8736 (24.8)	4709 (25.3)	0.0005	
6.3–10.8%	30,914 (29.7)	5325 (24.5)	8821 (30.8)	11,354 (32.3)	5414 (29.0)	<0.0001	
<6.3%	30,107 (28.9)	8210 (37.7)	8033 (28.1)	9014 (25.6)	4850 (26.0)	<0.0001	
Metro area							
Metro	88,903 (83.9)	17,586 (82.3)	23,503 (83.2)	30,071 (84.6)	17,743 (85.1)	<0.0001	3.2
Metro adjacent	10,741 (10.1)	2365 (11.1)	2988 (10.6)	3431 (9.7)	1957 (9.4)	<0.0001	
Non-metro adj	4293 (4.1)	921 (4.3)	1183 (4.2)	1394 (3.9)	795 (3.8)	0.002	
Rural	2053 (1.9)	484 (2.3)	574 (2.0)	651 (1.8)	344 (1.7)	<0.0001	
Charlson–Deyo Score							
0	75,577 (69.0)	16,048 (72.6)	20,329 (69.7)	24,832 (67.8)	14,368 (66.6)	<0.0001	0
1	25,184 (23.0)	4773 (21.6)	6788 (23.3)	8912 (24.3)	4711 (21.8)	0.036	
2	5818 (5.3)	947 (4.3)	1472 (5.0)	1988 (5.4)	1411 (6.5)	<0.0001	
3+	2898 (2.6)	340 (1.5)	588 (2.0)	893 (2.4)	1077 (5.0)	<0.0001	
Primary site							
Head of pancreas	51,671 (47.2)	10,477 (47.4)	13,786 (47.2)	17,218 (47.0)	10,190 (47.2)	0.598	0
Body of pancreas	17,308 (15.8)	3088 (14.0)	4420 (15.1)	6054 (16.5)	3746 (17.4)	<0.0001	
Tail of pancreas	14,650 (13.4)	2787 (12.6)	3808 (13.1)	4982 (13.6)	3073 (14.2)	<0.0001	
Pancreatic duct	342 (0.3)	80 (0.4)	86 (0.3)	119 (0.3)	57 (0.3)	0.152	
Islets of Langerhans	3 (0)	2 (0)	0 (0)	1 (0)	0 (0)	NA	
Other	2204 (2.0)	313 (1.4)	545 (1.9)	803 (2.2)	543 (2.5)	<0.0001	
Overlapping lesion of pancreas	9121 (8.3)	1750 (7.9)	2400 (8.2)	3127 (8.5)	1844 (8.6)	0.005	
Pancreas, NOS	14,178 (13.0)	3611 (16.3)	4132 (14.2)	4321 (11.8)	2114 (9.8)	<0.0001	
Stage							
1	5352 (5.1)	677 (3.5)	1204 (4.3)	1950 (5.5)	1521 (7.2)	<0.0001	2.9
2	15,681 (15.1)	2287 (11.8)	4199 (15.1)	5905 (16.5)	3290 (15.5)	<0.0001	
3	18,429 (17.7)	4116 (21.2)	5061 (18.3)	5826 (16.3)	3426 (16.2)	<0.0001	
4	64,630 (62.1)	12,350 (63.6)	17,260 (62.3)	22,074 (61.7)	12,946 (61.1)	<0.0001	
Treatment started, days from diagnosis	31.8 ± 31.4	32.3 ± 39.5	31.8 ± 31.1	31.3 ± 28.6	32.1 ± 27.2	<0.0001	3.7
Received chemotherapy	108,260 (98.9)	21,851 (98.9)	28,793 (98.8)	36,275 (99.1)	21,341 (99.0)	0.008	0.1
1-year mortality	70,406 (66.5)	16,195 (73.7)	20,373 (70.8)	22,032 (62.6)	11,806 (59.4)	<0.0001	3.3
2-year mortality	93,417 (89.7)	20,468 (93.3)	26,261 (91.5)	30,316 (87.1)	16,372 (87.5)	<0.0001	4.9

^1^ median (IQR), Spearman correlation. Comp. comm.—comprehensive community cancer center. For continuous variables, the “±” represents standard deviation. Int. network—integrated network cancer program, N.—North, S.—South, NA—Not applicable.

**Table 4:** Percent survival for non-surgical patients at different time intervals.

	2004–2007 (N = 22,108, 20.2%)	2008–2011 (N = 29,177, 26.7%)	2012–2015 (N = 36,625, 33.4%)	2016–2017 (N = 21,567, 19.7%)
3 months	82.0% (81.5–82.6%)	82.4% (82.0–82.8%)	84.7% (84.3–85.1%)	85.9% (85.4–86.3%)
6 months	58.0% (57.3–58.7%)	59.4% (58.8–60.0%)	66.4% (65.9–66.9%)	69.2% (68.5–69.8%)
9 months	39.2% (38.6–39.9%)	42.0% (41.4–42.6%)	50.7% (50.1–51.2%)	54.3% (53.6–54.9%)
12 months	26.4% (25.8–27.0%)	29.4% (28.9–29.9%)	38.1% (37.6–38.6%)	42.2% (41.5–42.9%)
18 months	12.7% (12.3–13.1%)	15.2% (14.8–15.7%)	21.9% (21.5–22.4%)	25.7% (25.1–26.3%)
24 months	6.8% (6.5–7.1%)	8.7% (8.4–9.0%)	13.7% (13.3–14.0%)	16.4% (15.9–17.0%)
Median survival (months)	7.2 (7.1–7.3)	7.6 (7.5–7.7)	9.2 (9.0–9.3)	10.1 (9.9–10.2)

## Data Availability

Data was obtained from the National Cancer Database, which provides participant user files (PUF) for disease specific sites. PUF are able to be obtained through an application process by investigators at Commission on Cancer accredited programs.

## Supplementary Materials

The following supporting information can be downloaded at: https://www.mdpi.com/article/10.3390/cancers15184464/s1, Table S1: Stage-Specific Hazard Ratios for death in surgical patients; Table S2: Mixed-effects Cox regression for patients undergoing surgery; Table S3: Stage-specific hazard ratios for death in non-surgical patients; Table S4: Mixed-effects Cox regression for non-surgical patients.
