# Supplementary material for: Changing Practice Patterns and Improving Survival for Patients with Pancreatic Ductal Adenocarcinoma
Source: Cancers (Basel). 2023 Sep 7;15(18):4464. doi: 10.3390/cancers15184464 (PMC10526129; doi:10.3390/cancers15184464)
Supplement: Supplementary file 1 [file cancers-15-04464-s001.zip › cancers-2480918-supplementary.pdf]

## Supplementary Materials

Table S1. Stage-Specific Hazard Ratios for death in surgical patients.

| Data subset | Effect of year of surgery | 95CI | p |
|---|---|---|---|
| Full cohort (all stages) | HR multiplies by 0.975 (drops 2.5%) per year | 0.971-0.981 | <0.0001 |
| Stage 1 only | HR multiplies by 0.96 (drops 4%) per year | 0.951-0.969 | <0.0001 |
| Stage 2 only | HR multiplies by 0.97 (drops 3%) per year | 0.967-0.977 | <0.0001 |
| Stage 3 only | HR multiplies by 0.95 (drops 5%) per year | 0.938-0.964 | <0.0001 |
| Stage 4 only | HR multiplies by 0.97 (drops 3%) per year | 0.955-0.980 | <0.0001 |

Table S2. Mixed-effects Cox regression for patients undergoing surgery.

| Factor | Hazard Ratio | Lower 95% CI | Upper 95% CI | P |
|---|---|---|---|---|
| **Year of Diagnosis** | 0.975 | 0.971 | 0.981 | <0.001 |
| **Facility type** | | | | |
| Community | REF | REF | REF | REF |
| Comp. comm. | 1.002 | 0.938 | 1.07 | 0.9517 |
| Academic | 0.917 | 0.857 | 0.981 | 0.0121 |
| Int. network | 0.985 | 0.919 | 1.056 | 0.6714 |
| **Facility Location** | | | | |
| New England | REF | REF | REF | REF |
| Middle Atlantic | 0.936 | 0.811 | 1.079 | 0.361 |
| South Atlantic | 1.123 | 1.042 | 1.209 | 0.0022 |
| East N. Central | 1.116 | 1.034 | 1.205 | 0.0049 |
| East S. Central | 1.166 | 1.06 | 1.283 | 0.0016 |
| West N. Central | 1.071 | 0.984 | 1.166 | 0.1133 |
| West S. Central | 1.064 | 0.96 | 1.18 | 0.2361 |
| Mountain | 1.026 | 0.93 | 1.131 | 0.6098 |
| Pacific | 1.059 | 0.977 | 1.146 | 0.1623 |
| **Age** | 1.009 | 1.008 | 1.01 | <0.001 |
| **Sex** | | | | |
| Female | REF | REF | REF | REF |
| Male | 0.939 | 0.92 | 0.958 | <0.001 |
| **Hispanic origin** | | | | |
| Yes | REF | REF | REF | REF |
| No | 0.905 | 0.858 | 0.955 | 0.0003 |
| **Race** | | | | |

| | | | | |
|---|---|---|---|---|
| White | REF | REF | REF | REF |
| Black | 0.999 | 0.964 | 1.036 | 0.9704 |
| Other | 0.923 | 0.87 | 0.979 | 0.0079 |
| **Insurer** | | | | |
| Medicare | REF | REF | REF | REF |
| Private | 0.932 | 0.906 | 0.96 | <0.001 |
| Medicaid | 1.117 | 1.061 | 1.177 | <0.001 |
| Other Government | 1.014 | 0.929 | 1.107 | 0.7602 |
| None | 0.963 | 0.901 | 1.03 | 0.2706 |
| **Income Quartile** | | | | |
| <$40,227 | REF | REF | REF | REF |
| $40,277-$50,353 | 0.968 | 0.932 | 1.006 | 0.1014 |
| $50,354-$63,332 | 0.942 | 0.902 | 0.984 | 0.0075 |
| >$63,333 | 0.885 | 0.839 | 0.934 | 0<0.001 |
| **Education Quartile** | | | | |
| >17.6% | REF | REF | REF | REF |
| 10.9%-17.5% | 1.036 | 0.995 | 1.079 | 0.0877 |
| 6.3%-10.8% | 1.04 | 0.989 | 1.094 | 0.1223 |
| <6.3% | 1.011 | 0.955 | 1.07 | 0.7063 |
| **Distance from hospital** | 0.986 | 0.976 | 0.996 | 0.0054 |
| **Metro area** | | | | |
| Metro | REF | REF | REF | REF |
| Metro adjacent | 1.062 | 1.019 | 1.107 | 0.0041 |
| Non-metro adj. | 1.024 | 0.966 | 1.087 | 0.4244 |
| Rural | 1.042 | 0.967 | 1.122 | 0.2839 |
| **Treatment started, days from diagnosis** | 1.017 | 1.009 | 1.025 | <0.001 |
| **Charlson/Deyo Score** | | | | |
| 0 | REF | REF | REF | REF |
| 1 | 1.103 | 1.077 | 1.129 | <0.001 |
| 2 | 1.228 | 1.174 | 1.285 | <0.001 |
| 3+ | 1.306 | 1.222 | 1.395 | 0<0.001 |
| **Primary Site** | | | | |
| Head of pancreas | REF | REF | REF | REF |
| Body of pancreas | 0.989 | 0.948 | 1.033 | 0.6254 |
| Tail of pancreas | 0.95 | 0.917 | 0.984 | 0.0041 |
| Pancreatic duct | 0.899 | 0.804 | 1.005 | 0.062 |
| Other | 1.031 | 0.932 | 1.141 | 0.5563 |
| Overlapping lesion of pancreas | 1.122 | 1.062 | 1.185 | <0.001 |
| Pancreas, NOS | 0.95 | 0.904 | 0.998 | 0.0429 |

| | | | | |
|---|---|---|---|---|
| **Stage** | | | | |
| 1 | REF | REF | REF | REF |
| 2 | 1.257 | 1.201 | 1.316 | <0.001 |
| 3 | 1.576 | 1.452 | 1.71 | <0.001 |
| 4 | 2.621 | 2.443 | 2.812 | <0.001 |
| **T stage** | | | | |
| TX | REF | REF | REF | REF |
| T0 | 0.474 | 0.375 | 0.599 | <0.001 |
| T1 | 0.688 | 0.611 | 0.776 | <0.001 |
| T2 | 0.907 | 0.815 | 1.01 | 0.0747 |
| T3 | 0.991 | 0.895 | 1.097 | 0.865 |
| T4 | 1.23 | 1.088 | 1.392 | 0.001 |
| **N Stage** | | | | |
| NX | REF | REF | REF | REF |
| N0 | 0.808 | 0.729 | 0.895 | <0.001 |
| N1 | 0.846 | 0.765 | 0.935 | 0.001 |
| **Nodes Examined** | 0.856 | 0.842 | 0.871 | <0.001 |
| **Nodes Positive** | 1.347 | 1.319 | 1.376 | <0.001 |
| **Received Chemotherapy** | 0.64 | 0.618 | 0.662 | <0.001 |

Abbreviations: Comp. comm.: comprehensive community cancer center, Int. network: integrated network cancer program, N.: North, S.: South.

Table S3. Stage-specific hazard ratios for death in non-surgical patients.

| | Effect of year of diagnosis | 95CI | p |
|---|---|---|---|
| Full cohort (all stages) | HR multiplies by 0.959 (drops 4%) per year | 0.957-0.962 | <0.0001 |
| Stage 1 only | HR multiplies by 0.96 (drops 4%) per year | 0.950-0.965 | <0.0001 |
| Stage 2 only | HR multiplies by 0.95 (drops 5%) per year | 0.950-0.959 | <0.0001 |
| Stage 3 only | HR multiplies by 0.96 (drops 4%) per year | 0.952-0.963 | <0.0001 |
| Stage 4 only | HR multiplies by 0.96 (drops 4%) per year | 0.960-0.965 | <0.0001 |

Table S4. Mixed-effects Cox regression for non-surgical patients.

| Factor | Hazard Ratio | Lower 95% CI | Upper 95% CI | P |
|---|---|---|---|---|
| **Year of Diagnosis** | 0.959 | 0.957 | 0.962 | <0.001 |
| **Facility type** | | | | |
| Community | REF | REF | REF | REF |
| Comp. comm. | 0.998 | 0.959 | 1.038 | 0.9089 |
| Academic | 0.875 | 0.832 | 0.921 | <0.001 |

| | | | | |
|---|---|---|---|---|
| Int. network | 0.953 | 0.912 | 0.997 | 0.0353 |
| **Facility Location** | | | | |
| New England | REF | REF | REF | REF |
| Middle Atlantic | 0.903 | 0.8 | 1.019 | 0.0973 |
| South Atlantic | 1.074 | 1.001 | 1.151 | 0.0456 |
| East N. Central | 1.061 | 0.991 | 1.136 | 0.0898 |
| East S. Central | 1.135 | 1.055 | 1.221 | 0.0007 |
| West N. Central | 1.036 | 0.962 | 1.115 | 0.3485 |
| West S. Central | 0.97 | 0.888 | 1.059 | 0.4952 |
| Mountain | 1.023 | 0.938 | 1.117 | 0.6078 |
| Pacific | 1.032 | 0.961 | 1.108 | 0.3913 |
| **Age** | 1.01 | 1.009 | 1.011 | <0.001 |
| **Sex** | | | | |
| Male | REF | REF | REF | REF |
| Female | 0.958 | 0.946 | 0.971 | <0.001 |
| **Hispanic origin** | | | | |
| No | REF | REF | REF | REF |
| Yes | 0.867 | 0.832 | 0.903 | <0.001 |
| **Race** | | | | |
| White | REF | REF | REF | REF |
| Black | 0.96 | 0.938 | 0.983 | 0.0008 |
| Other | 0.891 | 0.851 | 0.932 | <0.001 |
| **Insurer** | | | | |
| Medicare | REF | REF | REF | REF |
| Private | 0.95 | 0.933 | 0.968 | <0.001 |
| Medicaid | 1.09 | 1.053 | 1.128 | <0.001 |
| Other Government | 0.931 | 0.873 | 0.992 | 0.0269 |
| None | 1.092 | 1.038 | 1.149 | 0.0007 |
| **Income Quartile** | | | | |
| <$40,227 | REF | REF | REF | REF |
| $40,277-$50,353 | 0.957 | 0.932 | 0.983 | 0.0013 |
| $50,354-$63,332 | 0.944 | 0.916 | 0.972 | 0.0001 |
| >$63,333 | 0.909 | 0.872 | 0.948 | <0.001 |
| **Education Quartile** | | | | |
| >17.6% | REF | REF | REF | REF |
| 10.9%-17.5% | 1.038 | 1.005 | 1.073 | 0.0233 |
| 6.3%-10.8% | 1.027 | 0.984 | 1.072 | 0.2241 |
| <6.3% | 0.993 | 0.946 | 1.043 | 0.7887 |
| **Distance from hospital** | 0.973 | 0.963 | 0.982 | <0.001 |
| **Metro area** | | | | |

| | | | | |
|---|---|---|---|---|
| Metro | REF | REF | REF | REF |
| Metro adjacent | 1.058 | 1.029 | 1.088 | 0.0001 |
| Non-metro adj. | 1.052 | 1.01 | 1.095 | 0.014 |
| Rural | 1.057 | 0.997 | 1.121 | 0.0626 |
| **Treatment started, days from diagnosis** | 0.91 | 0.902 | 0.918 | <0.001 |
| **Charlson/Deyo Score** | | | | |
| 0 | REF | REF | REF | REF |
| 1 | 1.102 | 1.077 | 1.128 | <0.001 |
| 2 | 1.216 | 1.177 | 1.257 | <0.001 |
| 3+ | 1.439 | 1.374 | 1.507 | <0.001 |
| **Primary Site** | | | | |
| Head of pancreas | REF | REF | REF | REF |
| Body of pancreas | 0.994 | 0.976 | 1.012 | 0.5069 |
| Tail of pancreas | 1.153 | 1.128 | 1.179 | <0.001 |
| Pancreatic duct | 0.942 | 0.828 | 1.073 | 0.3699 |
| Other | 0.95 | 0.907 | 0.995 | 0.0285 |
| Overlapping lesion of pancreas | 1.044 | 1.017 | 1.071 | 0.0013 |
| Pancreas, NOS | 1.092 | 1.065 | 1.119 | <0.001 |
| **Stage** | | | | |
| 1 | REF | REF | REF | REF |
| 2 | 1.115 | 1.078 | 1.153 | <0.001 |
| 3 | 1.013 | 0.982 | 1.045 | 0.4066 |
| 4 | 1.671 | 1.62 | 1.723 | <0.001 |
| **Received Chemotherapy** | 0.497 | 0.434 | 0.569 | <0.001 |

Abbreviations: Comp. comm.: comprehensive community cancer center, Int. network: integrated network cancer program, N.: North, S.: South.
